# Holistic End-to-End Congestion Control for SAGIN-Integrated UAV Networks with Seamless Aerial–Terrestrial Integration

**DOI:** 10.3390/s26134105

**Published:** 2026-06-28

**Authors:** Liang Zong, Yun Cheng, Yi Yao

**Affiliations:** 1 College of Information, Hunan University of Humanities, Science and Technology, Loudi 417000, China; zongliang@huhst.edu.cn (L.Z.); yuncheng@huhst.edu.cn (Y.C.); 2 College of Information Science and Engineering, Shaoyang University, Shaoyang 422000, China

**Keywords:** Space–Air–Ground Integrated Networks (SAGINs), Unmanned Aerial Vehicle (UAV), heterogeneous network, transmission control

## Abstract

In Space–Air–Ground Integrated Networks (SAGINs), the inherent high bit error rate (BER) and prolonged propagation latency of satellite links, compounded by the highly dynamic topologies and multi-hop nature of Unmanned Aerial Vehicle (UAV) networks, present severe bottlenecks to end-to-end transport performance. To mitigate performance degradation within these heterogeneously converged SAGIN-UAV architectures, this paper proposes a SAGIN-enabled Adaptive End-to-End Congestion Control scheme. By exploiting the distinct transmission characteristics of long-delay, high-BER satellite links alongside terrestrial mobile multi-hop UAV networks, the Proposed Scheme optimizes data injection during the slow-start phase and introduces a high-precision loss differentiation mechanism during the congestion avoidance phase. This framework accurately distinguishes non-congestive losses (e.g., channel errors or topology switching induced by UAV mobility) from genuine buffer overflows. The simulation results demonstrate that the proposed adaptive scheme significantly reduces queuing delays at UAV nodes, accelerates transmission efficiency across multi-hop terminals, and enhances data throughput in high-latency environments. Ultimately, this scheme offers a resilient solution for optimizing end-to-end transport control and maximizing the overall transmission capability of SAGIN-enabled UAV networks.

## 1. Introduction

As the vision for sixth-generation (6G) mobile communications materializes, Space–Air–Ground Integrated Networks (SAGINs) have emerged as the foundational paradigm for constructing future communication architectures characterized by ubiquitous connectivity and global coverage [1,2,3]. Within this evolving landscape, Unmanned Aerial Vehicle (UAV) networks—leveraging their unique advantages of flexible deployment, high mobility, and a high probability of Line-of-Sight (LoS) transmission—are rapidly transitioning from isolated reconnaissance and surveillance platforms into critical aerial nodes. These nodes provide opportunistic hotspot coverage, emergency communication relaying, and large-scale Internet of Things (IoT) data aggregation. Particularly in complex scenarios where terrestrial infrastructure is compromised or entirely inaccessible, UAV networks embedded within the SAGIN architecture can effectively fill coverage blind spots, thereby facilitating dynamic synergy and complementarity between space and terrestrial resources.

However, seamlessly integrating UAV networks into the SAGIN framework to achieve efficient end-to-end data transmission still introduces severe physical- and network-layer challenges. Unlike relatively stable terrestrial optical fiber or microwave backhauls, the SAGIN environment exhibits extreme heterogeneity: satellite links are constrained by long propagation delays and high bit error rates (BERs), whereas low-altitude UAV links are heavily disrupted by highly dynamic topology changes, frequent channel blockages, and multi-hop relay instability [4,5,6]. In such hybrid scenarios, traditional Transmission Control Protocols—including TCP Reno, TCP Cubic, and even certain satellite-optimized TCP variants—frequently suffer from severe performance degradation. The fundamental flaw lies in their implicit assumption that “packet loss strictly equates to network congestion,” leaving them devoid of the fine-grained awareness needed to identify the root causes of packet drops. Consequently, when a packet is lost, the sender cannot distinguish whether the drop stems from genuine buffer overflows or transient link switching induced by high-speed UAV mobility, deep channel fading, or random bit errors on the satellite link.

This mechanistic vulnerability precipitates catastrophic throughput degradation. Upon encountering non-congestive packet losses, traditional protocols erroneously trigger standard congestion avoidance mechanisms and aggressively curtail their congestion window (cwnd), resulting in a precipitous drop in the transmission rate. In high Bandwidth-Delay Product (BDP) environments such as SAGINs, the window recovery process is exceedingly sluggish, leaving expensive spectrum resources underutilized for extended periods. Furthermore, topology reconfigurations triggered by the high-frequency mobility of UAV nodes often cause instantaneous routing disruptions. If the transport layer cannot rapidly adapt to this intermittent connectivity, end-to-end Quality of Service (QoS) fluctuations will intensify, occasionally culminating in connection failures.

Despite extensive academic efforts focused on physical-layer waveform design, MAC-layer scheduling, and network-layer routing optimization [7,8,9,10], there remains a distinct scarcity of Adaptive End-to-End Congestion Control mechanisms tailored specifically for heterogeneous SAGIN-UAV scenarios. Existing solutions either focus exclusively on single-domain optimizations (e.g., pure satellite or pure ad hoc networks)—making them difficult to migrate directly to integrated space–air–ground environments—or they rely on complex cross-layer signaling interactions that introduce prohibitive overhead and latency in highly dynamic networks.

To bridge these gaps, this paper proposes a SAGIN-Enabled Adaptive End-to-End Congestion Control scheme to overcome the limitations of traditional transport protocols. By comprehensively modeling the coupled transmission characteristics of long-delay, high-BER satellite links and highly dynamic, multi-hop UAV networks, this study systematically resolves the “spurious congestion” dilemma in heterogeneous integrated networks.

## 2. Related Work

### 2.1. Convergence of Space–Air–Ground Integrated Networks and UAV Networks

The rapid evolution of Marine Internet of Things (MIoT) systems has increasingly highlighted their critical role in maritime environments. While Low Earth Orbit (LEO) satellites are fundamental to MIoT operations by offering high reliability and low latency, satellite signals frequently suffer degradation over oceanic expanses due to the Earth’s curvature and the severe power constraints of MIoT devices. To overcome these connectivity limitations, Tesfaw [11] introduced a Space–Air–Sea Integrated Network (SASIN) leveraging Unmanned Aerial Vehicles (UAVs) as dynamic aerial relays. This architecture utilizes a Double Deep Q-Network (DDQN) to optimize three-dimensional UAV trajectories, thereby maximizing communication coverage while guaranteeing adequate data rates for MIoT terminals. Concurrently, Li [12] proposed a three-tier network architecture incorporating LEO satellites, High-Altitude Platforms (HAPs), and ground terminals, augmented by large language models (LLMs) and deep reinforcement learning (DRL). To mitigate challenges associated with frequent handovers and resource allocation across heterogeneous free-space optical and radio frequency (FSO/RF) links, their study introduced the LTQC-DAM algorithm. This method applies dynamic action masks to eliminate invalid explorations and adaptively tunes hyperparameters, demonstrating accelerated convergence, enhanced downlink transmission rates, and a 17.69% reduction in satellite handover frequencies.

The burgeoning low-altitude economy has further escalated the need for ultra-reliable UAV communications, a requirement that conventional terrestrial infrastructure struggles to satisfy given aerial mobility. Recognizing these limitations, Zhang [13] investigated Integrated Satellite–Terrestrial Networks (ISTNs) as a viable paradigm, emphasizing multidimensional resource management, multi-layer collaborative beamforming, and sensing-assisted communications to achieve ubiquitous wide-area coverage. To fortify computational capacities for 6G networks, Zhang [14] also proposed a SAGIN model-collaboration framework wherein LEO satellites function as edge nodes and terrestrial servers operate as cloud nodes. By jointly optimizing task allocation and resource scheduling, this framework significantly improves the inference accuracy of large-scale AI models when processing UAV-collected sensory data, effectively outperforming standalone cloud or distributed edge architectures.

Moreover, Zhou [15] conceptualized a Space–Air–Ground Integrated Low-Altitude Aerial Vehicle Network (SAG-LAAVN) grounded in a hyper-converged architecture that unifies data, operation, information, and communication technologies (DOICTs) tailored for intelligent transportation systems. Shi [16] introduced a satellite-UAV integrated model where UAV swarms establish Flying Ad hoc Networks (FANETs) utilizing LEO satellites as primary relays. Their simulations revealed that for swarms comprising fewer than 25 UAVs, integrating the Optimized Link State Routing (OLSR) and TCP protocols over 5G non-terrestrial networks yields optimal real-time performance and minimized packet loss. Arani [17] presented a comprehensive survey of learning algorithms within the SAGIN context, determining that 3D satisfaction-based learning algorithms consistently outperform alternative approaches in maximizing rewards across diverse deployment configurations. Additionally, Huynha addressed the challenge of reliable task offloading in SAGIN-assisted edge computing by deploying a multi-agent DRL framework, effectively doubling the reliable offloading rate compared to baseline strategies. Van [18] investigated the issue of reliable task offloading within Space-Air-Ground Integrated Networks (SAGINs) and proposed a deep reinforcement learning (DRL)-based solution. The proposed method achieved a two-fold increase in the reliable task offloading rate while significantly optimizing resource utilization across complex SAGIN scenarios.

### 2.2. Transmission Control in UAV Networks

Although the Transmission Control Protocol (TCP) remains the foundational standard for congestion control, its efficacy often deteriorates in highly mobile wireless environments, such as FANETs, due to inherent link volatility. In a comparative evaluation of TCP variants, Amponis [19] proposed “Swarm HTCP” (S-HTCP), which exhibited superior performance in sustaining reliable, real-time communications for UAV swarms. For large-scale UAV networks that demand precise status updates, An [20] introduced a Centralized Message Queuing Telemetry Transport (MQTT) framework (CMQC). This architecture substantially bolsters real-time data transmission efficiency, significantly reducing both Age of Information (AoI) and latency jitter when compared to conventional MQTT, TCP sockets, or CoAP implementations.

Within the scope of Beyond Visual Line-of-Sight (BVLOS) operations, Mohamed [21] validated the feasibility of commercial 4G LTE networks for the real-time remote control of UAVs. Empirical testing across distances up to 4200 km demonstrated that LTE networks can reliably extend operational ranges while maintaining an average control latency of less than 150 ms. Addressing the stringent bandwidth and power constraints typical of UAV swarms, Tang [22] leveraged semantic communication techniques to transmit exclusively control data that are critically relevant to tracking tasks, thereby dramatically reducing communication overhead. Employing Lyapunov drift-plus-penalty theory, the study derived a decentralized, closed-form solution for adaptive channel and tracking error management, offering robust mathematical proof for swarm stability and resource efficiency under dynamic fading conditions. To prolong the flight endurance of fixed-wing UAV formations, Wu [23] innovatively integrated aerodynamics into the design of a dual-function low-altitude wireless network (LAWN). This approach capitalizes on the wake-wash effect to autonomously guide UAV swarms into power-optimal “V”-shaped formations via local interactions. Concurrently, the study formulated a max-min optimization problem to guarantee formation stability and fairness, ultimately achieving a near-optimal beamforming strategy that effectively decouples the complex trade-offs among perception, communication, and control. Novitasari [24] evaluated the performance of the Real-Time Streaming Protocol (RTSP) and the User Datagram Protocol (UDP) for real-time drone video streaming within constrained network environments. The empirical findings indicate that UDP, owing to its lightweight and connectionless nature, yields lower latency and higher throughput, making it highly advantageous for real-time applications; however, it exhibits higher vulnerability to packet loss under unstable network conditions. Conversely, TCP-based RTSP guarantees reliable data transmission and a lower packet loss rate, albeit at the expense of increased latency and jitter, which potentially compromise real-time responsiveness.

To alleviate congestion in dense indoor UAV deployments, Haider [25] proposed a mobility-aware resource management framework that dynamically modulates transmission rates through an edge network controller, successfully preserving Quality of Service (QoS) even in high-density scenarios. He [26] introduced a Hop-by-Hop Redundancy-Assured Adaptive Coding (HHRAC) scheme to bolster transmission reliability. By integrating Cauchy matrix-based coding with queue length prediction for proactive congestion control, this technique markedly minimizes both transmission latency and end-to-end retransmissions. Regarding optimal protocol selection, Fei [27] evaluated a spectrum of TCP variants, concluding that although BBR demonstrates notable resilience to packet loss, no single protocol universally excels across all dynamic UAV mission profiles. Finally, Feng [28] applied an Advantage Actor-Critic (A2C) reinforcement learning framework to mitigate congestion at bottleneck links in UAV-assisted wireless networks, reporting an 18.9% enhancement in average throughput alongside a 2.8% reduction in latency relative to traditional Q-learning methodologies.

The remainder of this paper is organized as follows: Section 3 analyzes the SAGIN framework for UAV network models; Section 4 presents a novel SAGIN-Enabled Adaptive End-to-End Congestion Control for UAV Networks; Section 5 evaluates the proposed protocol through simulations; and Section 6 concludes the paper with a summary.

## 3. SAGIN Framework for UAV Networks

### 3.1. Link Dynamics and Transmission Challenges in Space–Air–Ground Integrated Networks

**Figure 1:**
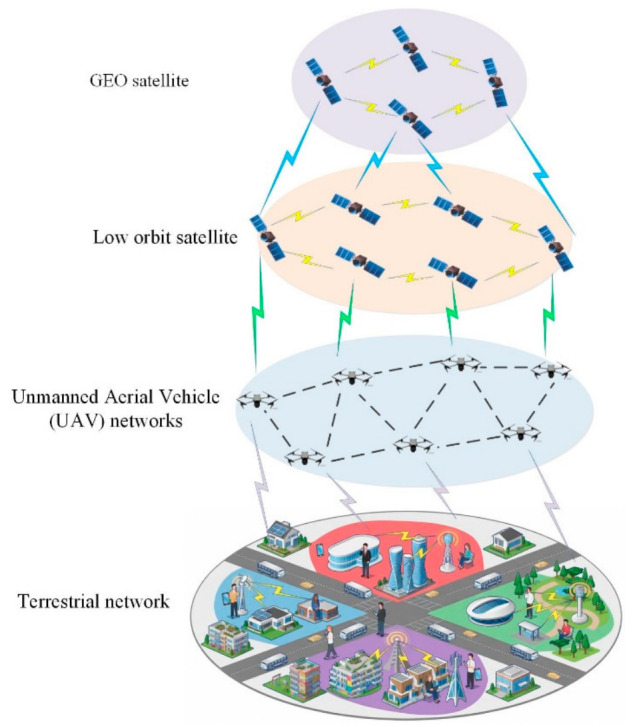
Multi-hop UAV network model in the SAGIN architecture.

The Space–Air–Ground Integrated Network (SAGIN) establishes a multi-tiered, three-dimensional communication architecture by unifying Geostationary Earth Orbit (GEO) satellites, Low Earth Orbit (LEO) satellites, Unmanned Aerial Vehicle (UAV) networks, and terrestrial infrastructures. As illustrated in Figure 1, this architecture is hierarchically organized into four functional layers:

- The top-tier GEO satellite layer provides wide-area coverage and core backbone backhaul.
- The middle-tier LEO satellite layer delivers low-latency regional relaying.
- The lower-tier UAV network layer acts as flexible, airborne mobile base stations or relay nodes characterized by high deployment agility.
- The bottom-tier terrestrial network encompasses dense urban infrastructure, vehicular networks, and user terminals.

In such a complex, heterogeneous ecosystem, the communication links exhibit exceptionally high dynamics. Specifically, the links between the UAV tier and the terrestrial tier, as well as the multi-hop intra-UAV links, are highly susceptible to severe shadowing by three-dimensional obstacles (e.g., high-rise buildings and dense foliage) and fast Doppler shifts induced by high-speed UAV mobility. Furthermore, the long propagation delays and periodic handovers characteristic of satellite links exacerbate the instability of end-to-end paths. These channel characteristics—defined by high dynamics, intermittent connectivity, and vast delay variations—render traditional transmission protocols based on static network assumptions incapable of sustaining efficient data delivery.

### 3.2. SAGIN Architecture Model for UAV Networks

Targeting future application scenarios such as smart cities, disaster relief emergency communications, and Vehicle-to-Everything (V2X), this paper constructs a multi-hop UAV network model embedded within the SAGIN framework (see Figure 1). In this model, the GEO and LEO satellite constellations constitute the space-based backbone, ensuring macro-connectivity for terrestrial blind spots or disaster zones that are otherwise unreachable. Concurrently, the UAV swarm forms an airborne self-organizing mesh network, functioning as High-Altitude Platforms (HAPs) to bridge satellite links and as aerial relays to connect ground-based users.

During the movement of terrestrial user equipment (e.g., autonomous vehicles, handheld terminals, and IoT sensors), uplinks must frequently hand over among terrestrial base stations, UAV nodes, and satellite links. When a UAV rapidly maneuvers behind structural obstructions or enters a signal shadow zone, the Line-of-Sight (LoS) link is instantaneously severed, causing bursty packet losses. Meanwhile, due to the integration of satellite segments, the end-to-end round-trip time (RTT) can fluctuate drastically, stretching from millisecond-level scales (via direct terrestrial/UAV connections) to hundreds of milliseconds (via satellite relays). Although this architecture significantly extends network coverage and enhances topological robustness, it imposes severe demands on transport-layer congestion control: the protocol must accurately distinguish link disruptions caused by physical blockages from genuine congestion caused by network overload while maintaining stable throughput across routing paths characterized by highly disparate mixed delays.

### 3.3. Limitations of Traditional TCPs in SAGIN-UAV Environments

The Transmission Control Protocol (TCP), the standard for internet transport, builds its core congestion control logic on the rigid paradigm that “packet loss directly implies network congestion.” This mechanism falls critically short in SAGIN-enabled UAV networks.

Taking TCP Reno as a baseline, it relies on packet loss events (indicated by timeouts or triple duplicate ACKs) to trigger a multiplicative decrease that halves the congestion window (cwnd). Under the SAGIN architecture, however, link disruptions caused by UAV mobility or environmental blockages are environmental norms rather than anomalies. When bursty packet drops occur on a UAV link due to transient obstructions, Reno erroneously misinterprets them as signs of network congestion, subsequently throttling the transmission rate aggressively. Given the high Bandwidth-Delay Product (BDP) inherent to satellite links, once the window is truncated, it takes an excessively long time to recover to the available capacity through the slow-start or congestion avoidance phases. This mismatch results in severe underutilization of expensive space-to-ground link resources.

Conversely, delay-based algorithms such as TCP Vegas attempt to proactively manage congestion by monitoring RTT variations. However, in SAGIN, RTT fluctuations originate primarily from the relative high-speed motion of satellite nodes and dynamic multi-hop routing reconfigurations rather than queue build-ups at intermediate buffers. The intermittent connectivity of UAV networks causes RTT measurements to experience abrupt spikes or enter unreachable states, which invalidates Vegas’s smooth delay-prediction mechanism and triggers severe sender-side oscillations or premature rate degradation.

Furthermore, conventional TCP protocols lack cross-tier topological awareness; they can neither leverage the redundant paths of GEO/LEO satellites for rapid failover nor adapt to the frequent topological reconfigurations inherent to UAV swarms. Therefore, designing an Adaptive End-to-End Congestion Control mechanism capable of sensing the holistic SAGIN state and accurately differentiating link failures from network congestion is paramount to unlocking high-performance transport in UAV-assisted networks.

## 4. SAGIN-Enabled Adaptive End-to-End Congestion Control for UAV Networks

### 4.1. Problem Formulation

In heterogeneous network environments, end-to-end performance is often bottlenecked by the maximum round-trip time (RTT) along the path. Consider a standard TCP connection where the congestion window, denoted as W(t) (in segments), evolves over time t. During the congestion avoidance (CA) phase, standard TCP adheres to an “additive increase” policy: upon the receipt of each acknowledgment (ACK), the window size is incremented by 1/W(t).

Approximating this discrete process with a continuous-time model, the growth rate of the window, dW/dt, can be expressed as the product of the ACK arrival rate ($\frac{1}{RTT}$) and the increment per ACK ($\frac{1}{W_{std}}$):

(1) $$\frac{dW_{std}}{dt}=\frac{1}{RTT}\cdot \frac{1}{W_{std}}=\frac{1}{W_{std}\cdot RTT}\;$$

Solving the differential Equation (1) yields the trajectory of the window’s evolution over time, $W_{std}(t)\approx \sqrt{2t/RTT}$. Consequently, the instantaneous sending rate B(t) is defined as B(t)=W(t)/RTT, which, upon substitution, results in:

(2) $$B_{std}\left(t\right)=\frac{1}{RTT}\sqrt{\frac{2t}{RTT}}=\frac{\sqrt{2t}}{RTT^{1.5}}$$

Equation (2) reveals an inherent flaw in standard TCP: the sending rate is proportional to $RTT^{-1.5}$. This implies that under identical packet loss rates and link capacities, the throughput of long-delay connections (e.g., satellite links or long-range UAV relays) is severely penalized, leading to unfair network resource allocation.

### 4.2. Design Objectives

The core objective of the Proposed Scheme is to eliminate the impact of the RTT on transmission performance, thereby achieving RTT-independent fairness. Specifically, for a connection with an arbitrary RTT, its sending rate $B_{New}(t)$ should equal the standard TCP rate $B_{ref}(t)$ of a reference short-delay connection (denoted as $RTT_{0}$, typically reflecting a standard terrestrial network RTT, e.g., 25 ms):

(3) $$B_{New}\left(t\right)=B_{ref}\left(t\right)=\frac{\sqrt{2t}}{RTT_{0}^{1.5}},\;\forall RTT\geq RTT_{0}$$

We define the normalized delay factor ρ as:

(4) $$\rho =\frac{RTT}{RTT_{0}},\;\rho \geq 1$$

When $RTT<RTT_{0}$, we set ρ=1 to maintain backward compatibility with short-delay connections. Based on the rate definition B(t)=W(t)/RTT, and combining Equations (3) and (4), we can derive the relationship between the target congestion window $W_{New}(t)$ and the reference window $W_{ref}(t)$:

(5) $$\frac{W_{New}(t)}{RTT}=\frac{W_{ref}(t)}{RTT_{0}}⟹W_{New}\left(t\right)=\frac{RTT}{RTT_{0}}W_{ref}\left(t\right)=\rho \cdot W_{ref}\left(t\right)$$

Equation (5) indicates that to maintain an equivalent sending rate, the congestion window of a long-delay connection must be scaled linearly by a factor of ρ.

Next, we derive the window growth formula that satisfies this objective. Differentiating both sides of Equation (5) with respect to time t:

(6) $$\frac{dW_{New}}{dt}=\rho \cdot \frac{dW_{ref}}{dt}$$

Substituting the standard TCP growth model for the reference connection (i.e., replacing RTT with $RTT_{0}$ and W with $W_{ref}$ in Equation (1)) into Equation (6) yields:

(7) $$\frac{dW_{New}}{dt}=\rho \cdot \frac{1}{W_{ref}\cdot RTT_{0}}$$

Using the relationship $W_{ref}=W_{New}/\rho$ to eliminate $W_{ref}$, we obtain the target differential equation in the continuous domain:

(8) $$\frac{dW_{New}}{dt}=\rho \cdot \frac{1}{\left(\frac{W_{New}}{\rho }\right)\cdot RTT_{0}}=\frac{\rho^{2}}{W_{New}\cdot RTT_{0}}$$

According to the discrete update rules, the window is updated across two phases: slow start and congestion avoidance. Congestion avoidance (CA): When cwnd≥ssthresh, for every non-duplicate ACK received, the window is updated as $cwnd\leftarrow cwnd+\rho^{2}/cwnd$. Slow start (SS): Standard TCP doubles its window every RTT during the slow start phase. To enable the Proposed Scheme to achieve identical exponential growth on the timescale of $RTT_{0}$, considering its longer actual RTT, accelerated growth is required. In practice, a simplified form or specific approximation is generally adopted, setting the increment per ACK during the slow start phase to $cwnd\leftarrow cwnd+(2^{\rho }-1)$.

### 4.3. Dynamic Feedback Gain-Based Congestion Window Adjustment Mechanism

To distinguish between random bit-error losses and congestion-induced losses in heterogeneous networks, we propose a window adjustment framework based on a dynamic feedback gain. Instead of relying on discrete mode switching, this framework continuously tunes control parameters according to the network backlog state, denoted as Φ(t).

#### 4.3.1. Network Backlog Potential

The network backlog potential at time t, Φ(t), is defined as the integral mapping of the current throughput error over the propagation delay. Deviating from traditional queue length estimations, we define it as the normalized deviation between the expected and actual rates:

(9) $$\Phi \left(t\right)=1-\frac{RTT_{min}}{RTT\left(t\right)}$$

where $RTT_{min}$ is the minimum observed round-trip time (representing the pure propagation delay), and RTT(t) is the currently measured round-trip time. The physical significance of Φ(t) is that it acts as a normalized indicator of the current link buffer occupancy.

Changes in physical topology can cause a shift in the baseline value of the RTT. The Proposed Scheme defines the network backlog potential as $\Phi (t)=1-RTT_{min}/RTT(t)$. To address path changes or satellite handovers, instead of treating $RTT_{min}$ as a global static value, the scheme designs it as a dynamic sliding-window update mechanism. When a route handover causes a step change in the propagation delay, the protocol will proactively reset $RTT_{min}$ if it continuously detects N RTT samples stabilizing within a new range. Thus, even if the RTT changes due to drone movement, Φ(t) accurately reflects the current link’s true “queue backlog” rather than changes in the topological physical distance, thereby maintaining the reliability of loss differentiation.

#### 4.3.2. Adaptive Control Operator

We define a nonlinear operator Ψ[Φ(t)] to dynamically generate the control gain based on the current network state. This operator is derived from a modified Sigmoid function to facilitate a smooth transition between “conservative adjustment” and “aggressive adjustment”:

(10) $$\Psi \left(\Phi \right)=\alpha \cdot \sigma \left(\kappa \left(\Phi -\Phi_{0}\right)\right)+\eta$$

where $\sigma (x)=1/(1+e^{-x})$ is the Logistic activation function; α, κ, and η are protocol parameters; and $\Phi_{0}$ represents the state decision bias point.

In our implementation, the parameters are configured as specific discrete mappings. However, to demonstrate generality, we express them in a piecewise limit form: As Φ(t)→0 (light load), $\Psi \to \psi_{rand}$ (gain in the random loss regime). As Φ(t)→1 (heavy load), $\Psi \to \psi_{cong}$ (gain in the congestion loss regime).

#### 4.3.3. Window Reduction Dynamics

Traditional AIMD models execute a fixed halving operation upon the occurrence of a packet loss event $E_{loss}$. The mechanism proposed in this paper introduces a state-dependent reduction factor Λ(Φ), restructuring the multiplicative decrease (MD) step as:

(11) $$W\left(t^{+}\right)=W\left(t^{-}\right)\cdot \Gamma_{md}\left(\Phi \left(t^{-}\right)\right)$$

where the reduction mapping function $\Gamma_{md}$ is defined as:

(12) $$\Gamma_{md}\left(\Phi \right)=\frac{1}{2}+\left(\frac{4}{5}-\frac{1}{2}\right)\cdot H\left(\beta -N\left(\Phi \right)\right)$$

Here, H(⋅) is the Heaviside step function, and $N(\Phi )=\Phi \cdot W(t^{-})/(1-\Phi )$ represents the implicitly estimated queue backlog. If the system detects that the backlog N is less than the threshold β at the time of packet loss (i.e., H=1), the reduction factor is set to 0.8; otherwise, it defaults to the standard 0.5. Formally, this manifests as a state-gated reduction operator.

The above process models the AIMD (Additive Increment, Multiplicative Increment, Subtractive Increment) process as a nonlinear dynamic system. The introduced reduction mapping function $\Gamma_{md}\left(\Phi \right)$ serves as a negative feedback loop through implicit queue backlog N(Φ). Theoretical analysis shows that as long as 0 < $\Gamma_{md}$ < 1 and the growth operator Θ(Φ) is Lipschitz continuous, the eigenvalues of the system’s Jacobian matrix will have negative real parts, thus ensuring that the system converges to a unique stable attractor, guaranteeing stability and fairness in bandwidth sharing under macroscopic network conditions.

#### 4.3.4. Smooth Growth Operator

To suppress the severe oscillations of traditional Additive Increase (AI) at the bottleneck, we introduce a state-modulated clock factor Ω(Φ). The differential equation for window growth is defined as:

(13) $$\frac{dW(t)}{dt}=\frac{1}{RTT(t)}\cdot \Theta \left(\Phi \left(t\right)\right)$$

where the modulation function Θ(Φ) is defined as:

(14) $$\Theta \left(\Phi \right)=1-\frac{1}{2}\cdot I\left(\Phi \geq \Phi_{th}\right)$$

I(⋅) represents the indicator function. When the backlog potential Φ exceeds the threshold $\Phi_{th}$ (corresponding to N≥β), the value of Θ transitions from 1 to 0.5.

Corollary (Growth Rate): This implies that in the continuous-time domain, the growth rate of the congestion window is automatically reduced by 50% under high-load conditions. When the bottleneck link is saturated, the growth step per RTT is reduced from 1 Maximum Segment Size (MSS) to 0.5 MSS, thereby enabling the system to reside near the optimal operating point for a more extended duration.

The continuous-time model derivative $dW/dt=\rho^{2}/(W_{New}\cdot RTT_{0})$ in practical implementation relies on the smoothed RTT (SRTT) rather than instantaneous RTT sampling. Furthermore, the dynamic feedback gain Φ(t) employs a nonlinear operator Ψ(Φ) based on the Logistic function. This operator is designed with “low-pass filtering” characteristics, effectively absorbing and tolerating millisecond-level instantaneous RTT jitter, preventing frequent and unnecessary switching of the congestion control state machine.

## 5. Simulation Analysis

### 5.1. Simulation Model

This study constructs a high-fidelity Space–Air–Ground Integrated Network (SAGIN) heterogeneous cascaded model. The model comprises three heterogeneous segments: a terrestrial/cloud core network hosting the source server, a Geosynchronous Orbit (GEO) satellite backbone relay link, and an edge-access multi-hop cluster consisting of five Unmanned Aerial Vehicles (UAVs). Within the UAV cluster, a chain topology Mobile Ad hoc Network (MANET) is employed for multi-hop data forwarding. The data receiver can dynamically switch between UAV nodes from the 1st to the 5th hop to simulate varying depths of edge coverage. The link bandwidth is 1.54 Mbps, the routing buffer is 16 Mbytes, and the router nodes use the DropTail queuing strategy. In the simulation, the size of all packets is fixed at the standard 1500 bytes (MTU = 1500 B).

The topology incorporates two highly disruptive physical network characteristics, which represent core challenges in current 6G communications and channel estimation: 1. Ultra-long Propagation Delay and Large Bandwidth-Delay Product (BDP): The GEO satellite is positioned approximately 36,000 km from Earth, resulting in a base round-trip time (RTT) for the satellite–terrestrial link that typically exceeds 500 ms. This extreme latency severely inhibits the slow start and congestion avoidance processes of traditional TCPs. 2. Highly Dynamic Wireless Channels and Space-Channel Fading: While the satellite–terrestrial link is affected by atmospheric absorption, rain fade, and ionospheric scintillation, the UAV cluster links are constrained by Doppler shifts and highly dynamic topologies. Despite the use of advanced channel estimation and Forward Error Correction (FEC) at lower layers, the transport layer inevitably encounters residual Random Bit Errors (BERs).

Four representative end-to-end congestion control schemes are selected for performance benchmarking: Scheme 1 (TCP Reno [29]: a classic loss-driven protocol), Scheme 2 (TCP Veno [30]: a protocol with wireless random loss discrimination), Scheme 3 (TCP Hybla [31]: a protocol designed with RTT compensation for satellite links), and the Proposed Scheme. The primary performance metric is the end-to-end network throughput (measured in packets/s). To evaluate protocol robustness across different channel degradation gradients, the physical-layer BER of the satellite–terrestrial link is set to five logarithmic scales: $1\times 10^{-5}$, $1\times 10^{-6}$, $1\times 10^{-7}$ and a near-error-free environment at $1\times 10^{-8}$.

### 5.2. Analysis of “Pseudo-Congestion” Collapse in Extreme Channel Conditions ($BER=1\times 10^{-5}$)

**Figure 2:**
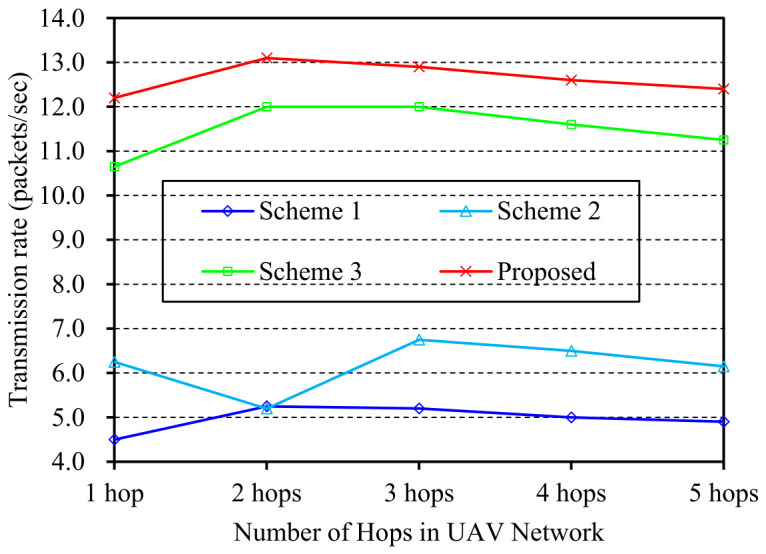
Transmission rate of the link at high BER.

When the satellite–terrestrial link faces the harshest electromagnetic environment ($BER=1\times 10^{-5}$), a high volume of packets experiences bit flips at the physical layer and is discarded by the MAC layer checksum, triggering dense random packet losses, as seen in Figure 2.

The experimental data reveal that across the entire 1-to-5 hop topology, both Scheme 1 and Scheme 2 suffer from severely degraded performance. In the 1-hop gateway access scenario, Scheme 1 achieves a throughput of only 4.5 packets/s, while Scheme 2 reaches 6.25 packets/s. Even extending to 5 hops, their performance remains at a marginal 4.9 and 6.15 packets/s, respectively.

This “performance cliff” stems from “TCP Blindness.” At a $10^{-5}$ BER, the congestion control state machine of Scheme 1 is frequently interrupted by random losses. Although no actual router buffer overflow occurs, Scheme 1 misinterprets every wireless loss as a congestion signal, repeatedly triggering multiplicative decrease to halve the congestion window (cwnd). This “pseudo-congestion” prevents the window from effectively opening over long RTT links. While Scheme 2 attempts to utilize network backlog to distinguish random loss, the high-frequency bit errors interfere with its $RTT_{min}$ sampling, causing state machine oscillations and rendering its discrimination algorithm ineffective.

In contrast, Scheme 3 maintains 10.65–12.0 packets/s due to its inherent resilience to long delays. However, the Proposed Scheme exhibits dominant performance, reaching 11.9, 12.9, 12.9, 12.4, and 12.2 packets/s across 1 to 5 hops. Compared to the Scheme 1 baseline, the Proposed Scheme achieves a 164.4% performance gain in 1-hop scenarios and maintains over 140% stable gain in multi-hop scenarios. This demonstrates that by optimizing channel state awareness, the Proposed Scheme accurately filters out independent losses caused by wireless fading and maintains a sufficient scale of in-flight packets despite the 500 ms satellite RTT.

### 5.3. “Hop-Count Penalty” and Spatial Reuse Interference in Moderate Channel Degradation ($BER=1\times 10^{-6}∼1\times 10^{-7}$)

**Figure 3:**
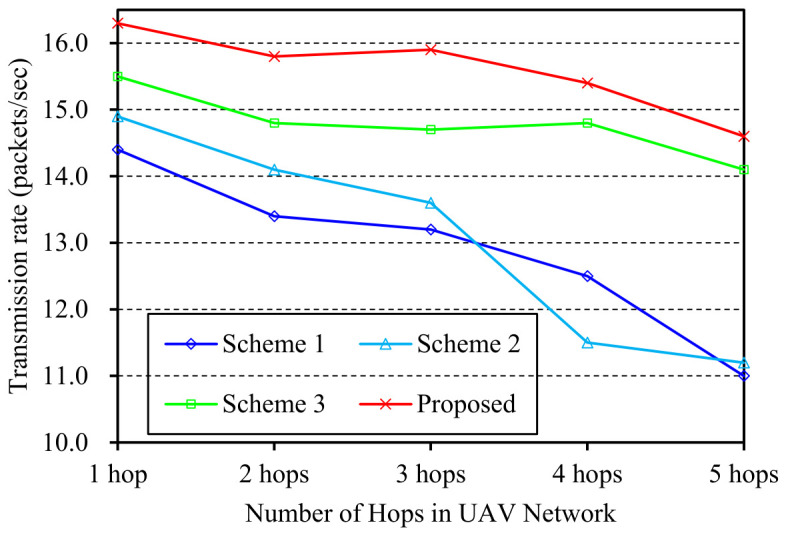
Transmission rate of the link at middle BER ($BER=1\times 10^{-6}$).

**Figure 4:**
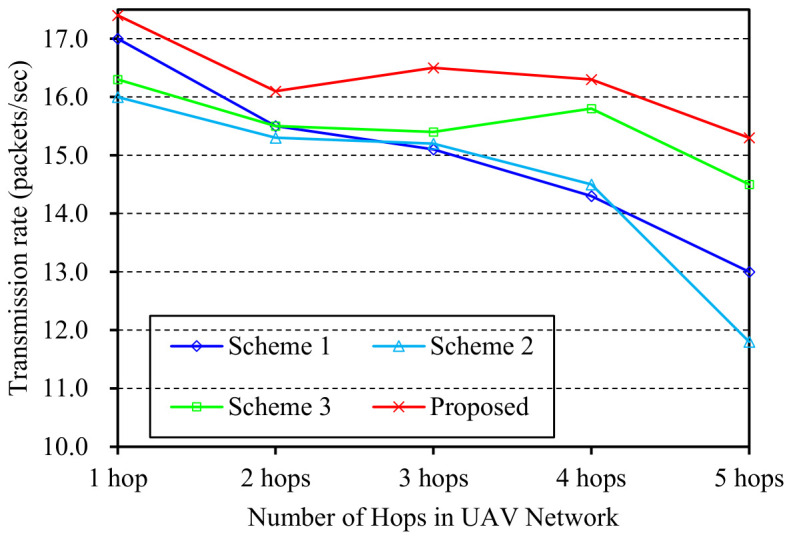
Transmission rate of the link at middle BER ($BER=1\times 10^{-7}$).

As the channel quality improves to the $10^{-6}$ and $10^{-7}$ levels due to FEC, all schemes show significant performance jumps. At these levels, the results show “numerical convergence”—for instance, at 1 hop, Scheme 1 stabilizes at 14.4 packets/s, Scheme 2 at 14.9 packets/s, and the Proposed Scheme at 16.1 packets/s, as seen in Figure 3 and Figure 4.

This indicates that once the BER falls below $10^{-6}$, random loss is no longer the primary bottleneck. The core network challenge shifts to long propagation delays and MAC-layer contention/queuing delays inherent in multi-hop UAV relays.

The Proposed Scheme accurately characterizes the “Hop-count Penalty” inherent in MANETs, with the throughput gradually decreasing from 16.1 packets/s (1 hop) to 14.5 packets/s (5 hops). In a chain topology, adjacent nodes share the same spectrum, and the CSMA/CA-based MAC layer inevitably suffers from “Hidden Terminal” and “Exposed Terminal” issues, alongside intense intra-flow interference. Each hop adds MAC-layer backoff delay and non-linear growth in buffer occupancy.

Despite these complexities, the Proposed Scheme remains optimal. A notable counter-example is Scheme 2’s failure at 4 hops, where the throughput plummeted from 13.6 to 11.5 packets/s. This was caused by the jitter reaching a threshold that misled Scheme 2’s bandwidth estimation model. The Proposed Scheme’s smoother adaptive thresholding effectively absorbed these topological oscillations.

### 5.4. “Deep Queue Catastrophe” and RTO Collapse in Near-Error-Free Environments ($BER=1\times 10^{-8}$)

**Figure 5:**
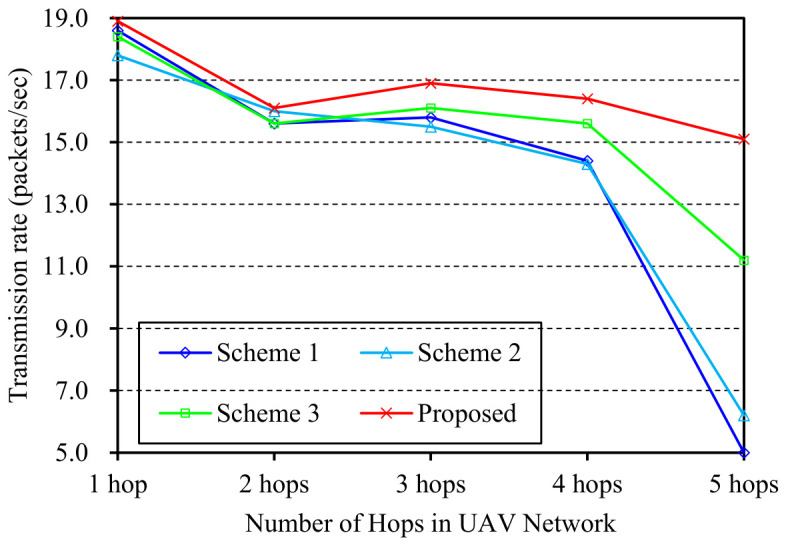
Transmission rate of the link at low BER ($BER=1\times 10^{-8}$).

In nearly transparent channel conditions ($BER=10^{-8}$), the performance is characterized by convergence at low hop counts and severe divergence at high hop counts. At 1 hop ($10^{-8}$), Scheme 1 benefits from its aggressive Additive Increase, reaching 18.5 packets/s, while the Proposed Scheme peaks at 18.9 packets/s, as seen in Figure 5.

However, a “throughput avalanche” occurs at 5 hops with $BER=10^{-8}$. While all protocols perform well up to 4 hops, at the 5th hop, Scheme 1 and Scheme 2 crash to 5.0 and 6.2 packets/s, respectively—a nearly 73% drop for Scheme 1.

This collapse is triggered by spurious RTO (Retransmission Timeout) events induced by Bufferbloat in multi-hop deep queues. In error-free states, TCP sources aggressively expand cwnd, flooding the 5-relay UAV chain. Due to the “Funneling Effect” and channel contention, intermediate UAV nodes process data much slower than the gateway injects it, leading to extreme queuing delays.

According to the Jacobson/Karn algorithm, RTO is calculated as:

(15) RTO=SRTT+4×RTTVAR

When queuing delay causes the RTT to spike beyond the RTO threshold, Scheme 1 and Scheme 2 misinterpret the delay as a total network failure. They respond by resetting cwnd to 1 MSS and re-entering slow start. This leads to a Deadlock cycle of “Backlog → Spurious Timeout → Window Reset,” causing total throughput collapse.

The Proposed Scheme demonstrates remarkable architectural resilience, maintaining a robust 15.1 packets/s at 5 hops—outperforming the collapsed Scheme 1 by 202%. It achieves this through a high-order flow control mechanism that dynamically estimates both the “Bottleneck Bandwidth” and “Minimum Path RTT.” By detecting early signs of non-linear queue expansion, it implements “Soft Braking” to limit injection rates, ensuring the load matches the UAV network’s physical capacity. This proactive strategy prevents buffer overflow and timer collapse, reaching a Pareto optimal equilibrium between high throughput and low queuing delay.

### 5.5. Discussion

Simulation across five BER magnitudes and five hop depths proves that in the heterogeneous SAGIN environment, traditional single-mode congestion control is obsolete. The Proposed Scheme breaks the two-dimensional design framework of classic protocols. By integrating an improved RTT scaling algorithm with a jitter-resistant wireless state discriminator, it successfully mitigates “pseudo-congestion” in noisy channels ($10^{-5}$) and prevents “deep queue collapse” in multi-hop topologies (5 hops). This provides a solid theoretical and engineering foundation for high-reliability, high-throughput 6G transport protocols.

While this study rigorously evaluates the transport-layer performance under extreme BER and multi-hop queuing delays—which effectively abstract the bursty packet loss and RTT spikes caused by SAGIN mobility—explicit modeling of 3D orbital dynamics and satellite handovers was not conducted. Future work will focus on cross-layer evaluations incorporating high-fidelity orbital mobility models to further validate the protocol’s transient response during macro-topological reconfigurations.

## 6. Conclusions

This paper has addressed the critical challenge of end-to-end transmission inefficiency within Space–Air–Ground Integrated Network (SAGIN)-enabled Unmanned Aerial Vehicle (UAV) networks. The pronounced heterogeneity of these architectures—characterized by the extensive propagation delays of satellite links, elevated bit error rates (BERs), and the highly dynamic, multi-hop nature of UAV relays—renders conventional TCP congestion control mechanisms largely ineffective. The fundamental flaw in legacy protocols is their rigid assumption that packet loss strictly signifies network congestion, a misinterpretation that triggers severe performance degradation when losses actually stem from non-congestive anomalies such as channel fading or rapid UAV mobility.

To systematically resolve these bottlenecks, we proposed a SAGIN-Enabled Adaptive End-to-End Congestion Control scheme. This framework introduces a comprehensive dual-layer optimization strategy. First, it incorporates an RTT-independent fairness mechanism that scales the congestion window according to a normalized delay factor. This ensures that long-delay satellite links can achieve throughput performance commensurate with terrestrial connections, without sacrificing network stability. Second, the protocol integrates a dynamic feedback gain controller that continuously evaluates the network backlog potential. By leveraging a non-linear, Sigmoid-based operator, the proposed scheme accurately distinguishes between random bit-error losses and authentic buffer overflows. This discriminatory capability effectively eliminates the spurious congestion avoidance behaviors that severely handicap traditional transport protocols.

Extensive simulations conducted across a heterogeneous SAGIN testbed—spanning from severely degraded, high-BER environments ($10^{-5}$) to near-error-free conditions—robustly validate the superiority of the proposed scheme. The empirical results confirm that the proposed scheme successfully mitigates “pseudo-congestion” collapse in noisy channels while preventing “deep queue catastrophes” in multi-hop UAV deployments. Notably, within a 5-hop UAV relay topology under extreme network conditions, the proposed scheme sustained a high-throughput transmission rate of 15.1 packets/s, outperforming the standard TCP Reno baseline by over 202%.

Ultimately, this research delivers a resilient transport-layer solution that significantly advances the transmission capabilities and reliability of SAGIN-UAV networks. By intelligently adapting to both fluctuating channel states and dynamic network loads, the Proposed Scheme guarantees the high spectral efficiency and stable connectivity requisite for emerging 6G-oriented aerial–terrestrial integrated services.

## Data Availability

The original contributions presented in this study are included in the article. Further inquiries can be directed to the corresponding authors.
